# The Impact of Stress and Distraction on Bag-Valve-Mask Ventilation Performance

**DOI:** 10.7759/cureus.84542

**Published:** 2025-05-21

**Authors:** Grant H McDaniel, Scott Pappada, Zakaria Alyosif, Solomon Teye-Lartey, Mohamad Moussa

**Affiliations:** 1 Medical Education, University of Toledo College of Medicine and Life Sciences, Toledo, USA; 2 Bioengineering, University of Toledo, Toledo, USA; 3 Exercise and Rehabilitation Sciences, University of Toledo, Toledo, USA; 4 Emergency Medicine, University of Toledo College of Medicine and Life Sciences, Toledo, USA

**Keywords:** feedback devices, medical education, physiologic monitoring, simulation, stress

## Abstract

Objectives: Stress and distraction are intrinsic to the emergency department (ED). These factors can invoke a physiologic response, potentially leading to performance decrements in life-saving procedures like bag-valve-mask (BVM) ventilation. The aim of this study is to use a simulation scenario for two reasons: (1) to evaluate how stress and distraction impact emergency medicine (EM) residents’ physiology; (2) to evaluate how these factors impact BVM ventilation performance. Our hypothesis is that both factors will cause performance decrements, with stress causing increased ventilations and distraction causing disengagement and reduced ventilations.

Methods: Four EM residents were subjected to a simulation scenario comprised of three phases: (1) baseline (no stress or distraction); (2) stress (increased alarm/alerts); and (3) distraction (multiple disruptions caused by simulation center staff).

Throughout the simulation each subject wore a physiologic monitor that recorded their heart rate (HR) and electrodermal activity (EDA). A high-fidelity manikin was utilized to record the BVM ventilation performance. The manikin recorded the number of ventilations delivered, duration of the ventilation and the peak inspiratory pressure (PIP) of each ventilation. To standardize physiological measures deviations of each data source relative to an algorithm-derived baseline/resting state was calculated.

Results: The average number of ventilations per minute during phase (1) was 12.5, (2) was 16, and (3) was 11.9. The average PIP (cmH_2_O/mL) delivered during phase (1) was 15.5, (2) was 24.4, and (3) was 22.3. Percent deviation from baseline for HR ranged from 16.9% to 38.4%, with three out of four EM residents having greater change during distraction. EDA data showed a 25-190.3% deviation from base line, with three out of four EM residents having the greatest deviation during the distraction phase.

Conclusion: Stress and distraction both had an impact on subject’s physiologic stress response and BVM performance. While the stress phase caused the highest performance decrements, the distraction phase was associated with the more significant physiologic response and self-reported impact.

## Introduction

Background

Stress and distractions are synonymous with emergency medicine (EM). Life-or-death situations, fear of mistakes, alarms and disruptions are common in the emergency department (ED) [1]. Medical device alarms are a known source of stress due to their frequency, association with a patient in need, or being a nuisance (i.e., false alarm). An observational study found ED vital monitors actuated on average 19.5 timers per hour [2]. EM physicians reported that their workflow was interrupted by others an average of 12.5 times per hour [3]. Stress and distraction have been shown to lead to delays in interpreting information, subsequent decision making, the ability to perform medical procedures and increased prescribing error [4-7]. They are commonplace in the ED and can negatively impact physicians and their ability to perform patient care.

Simulation-based training (SBT) is often employed to help train healthcare professionals (HCPs) to deal with stress and distraction. SBT has been shown to improve trainees’ knowledge, skills, behavior, and potentially patient outcomes [8]. Simulations that incorporate use of high-fidelity manikins coupled with debriefing (post-scenario performance feedback) have been shown to have a positive association to both skill and knowledge retention, compared to lower fidelity simulations without feedback on performance [9,10]. To provide granular and focused feedback to learners, it is necessary to collect a holistic set of data from the simulation environment (e.g., data from human patient simulators or manikins), learner (functional state changes that reflect their experience and proficiency, e.g., stress), and instructor (e.g., evaluations or performance data recorded during observation). This feedback can be collected with wearable physiologic monitors which provide more actionable data for the instructor and learners.

Over the past decade, innovations in wearable physiologic monitors has led to an increase in their use for studying factors impacting HCP performance. They have been used to study the impacts of stress on sleep and burnout [11-13]. Recently, researchers have been implementing wearables into medical simulation training [14,15]. Modern wearables provide the ability to gather further granular details about how a simulation is impacting a subject by providing electrodermal activity (EDA) and heart rate (HR). A sympathetic stress response is signaled by an increase in HR and EDA [14]. The use of physiologic wearables in conjunction with high-fidelity feedback manikins offers an innovative way to observe the performance of health care providers during simulation and stress.

The practice of EM requires the employment of various skills that are susceptible to decrement based on the level of stress and distraction present within the patient care environment. Bag-valve-mask (BVM) ventilation is a skill often required during critical situations to stabilize a patient for further resuscitation. The American Heart Association (AHA) provides guidelines for the use of a BVM, recommending 10 ventilations per minute with each delivered over one second, for a patient who is in respiratory failure with a pulse [16]. HCPs have been shown to routinely exceed these guidelines in both the simulation and the clinical environment [17-20]. In addition to hyperventilation/overventilation with too much volume or pressure (>10 mL/kg of tidal volume or peak inspiratory pressure (PIP) >20 cmH_2_O/mL) is associated with barotrauma and gastric distention, both increasing mortality [17-21]. The skill of BVM ventilations is highly susceptible to decrement based on the subject, the environment they are in, and their perceived stress level [22].

There is a growing body of research studying the stress response and its association with performance. A study looking at military personnel used various biomarkers and HR variability to identify modifiable and nonmodifiable stressors that impact performance [23]. Others have also thought to categorize various stress inducing factors and their relation to task engagement and performance [24-26]. A recent study had EM residents participate in simulated scenarios with three predetermined stressors. During the simulation, the EM residents wore HR monitors and filled out a questionnaire at the conclusion of the scenario. Their results showed that interpersonal stressors had the greatest impact [26].

SBT has made significant progress over the last several decades. Medical training programs often integrate dynamic clinical scenarios, high-fidelity manikins, real-time feedback, and some are even incorporating wearable physiologic monitors. Our work aims to use a multidimensional approach, utilizing physiologic monitors, real-time feedback, manikins, and subjects’ perceived stress to analyze the effects of stress and distraction on them.

Importance

The impact of stress and distraction on a HCP’s ability to delivery care can have detrimental effects on patient outcomes. One evolving way to mitigate this effect is SBT, which is becoming more common in the education and training of HCPs. While this training has proven benefits, the lack of a standardized and performance-driven approaches leaves a large area for further research and innovation. Our project touches on this training method to teach and learn about the impact of stress and distraction in BVM ventilation. 

Goals

The goals are to build and test a model that can be reproduced and further studied, and to better understand how stress and distraction effect performance. The data gathered from real time feedback devices have the potential to improve SBT and ultimately patient care in several ways. On an individual level, a performance profile can be generated from the feedback. That data can then be used to generate specific simulations targeted at the participant weaknesses. As the participant progresses, their results can be compared to baseline to determine response. Implemented on a program level, the data can be analyzed to look for trends that uncover deficiencies in education and offer areas for improvement. Globally, the results can help inform the medical community of the impact of stress and distraction on performance leading to further research into ways to mitigate their effects.

## Materials and methods

Study design

The study aims to employ a simulation for two specific purposes: (1) to evaluate how stress and distraction impact EM residents’ physiology; (2) how these factors impact BVM ventilation performance. Our hypothesis is that both factors will cause performance decrements, with stress causing increased ventilations and distraction causing disengagement and reduced ventilations. To investigate our hypothesis our study included three phases: (1) simulation-scenario development, (2) technology deployment, and (3) data analysis. 

Simulation-scenario development

**Figure 1:**
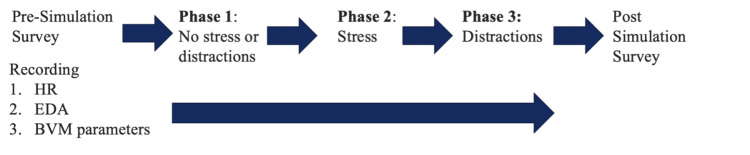
Three-phase simulation scenario design

A three-phase simulation scenario was developed that incorporated various stressors and distractions into the clinical workflow of an EM physician during a patient scenario requiring BVM ventilation (Figure 1). Throughout the simulation, subjects were required to perform BVM ventilations per AHA standards for a patient not breathing but who has a pulse (one ventilation delivered over one second every six seconds). Subjects were instructed to familiarize themselves with equipment prior to the start of the simulation. Phase 1 of the simulation (baseline) was two minutes in duration without a stimulus to establish baseline metrics for a non-stressful and distraction free environment. Afterwards, Phase 1 subjects were asked to exit the room and await further instruction. Phase 2 was three minutes in duration and involved induction of stressors via instituting a continuous loop of alarms from ventilators, monitors, intravenous pumps, along with other alerts from cell phones and overhead pages. Phase 3 was also three minutes in duration and involved several actors simulation center staff inducing distractions to the simulation participant via several scripted conversation. One of the conversations in which patient care was interrupted was by someone entering the room to inform the team about an incoming high patient load resultant from a mass casualty incident. Simulation center staff were instructed to become louder and to increase aggression as the scenario progressed. 

Simulations were conducted at the Lloyd A. Jacobs Interprofessional Immersive Simulation Center on the campus of University of Toledo Medical Center. The simulations of this project took place in a high-fidelity simulated environment representative of an ED and were facilitated by trained simulation staff who regularly run complex medical simulations.

BioMedical Internal Review Board approval was obtained to conduct the research study (Approval no. 301459). EM residents were recruited via email and in person recruiting events. Incentives utilized for participation were free food and a $25 Amazon gift card. EM residents were the selected learner population as they had required AHA training and were familiar with BVM skills and equipment. Furthermore, EM residents represent an ideal target group to participate because they frequently are required to perform interventions in a stressful and distracting environment. A total of 13 subjects registered however only six presented to their appointments to complete the simulation. Moreover, simulation participants included three postgraduate year (PGY)-2 and three PGY-3 residents. Two of the six subjects enrolled to the study were excluded from analysis due to technical difficulties and error in data recording. The study preparation recruitment and simulation session took place from February 1, 2022, and March 1, 2023. 

Technology deployment

A primary goal of this study was to collect data from the learner and simulation environment. Prior to collecting data during the developed simulations, technology needed to be setup and deployed within the targeted simulation environment. A commercially available E4 wearable device (Empatica, Cambridge, USA) was deployed to monitor HR and EDA of simulation participants. This serves to collect objective learner-level data with respect to their perceived stress and experience in each simulation. The monitoring of HR and EDA provides noninvasive monitoring of the sympathetic response and is an established method for doing so [11-15,23,24,26]. Performance of simulation participants with respect to BVM ventilations was evaluated via measuring PIP, ventilation rate, and duration of ventilation [17-21]. To measure this environment-level data, a Gumard Trauma Hal manikin was utilized. Additional learner-level data was collected via a post-event survey delivered after the simulation via Qualtrics. This provided a quantitative self-evaluation to determine which phase of the developed simulation each participant considered the most impactful.

Data analysis 

All measurements excluding the surveys were grouped into three categories: (1) baseline; (2) stress phase; and (3) distraction phase. The stress and distraction phase were then compared to the baseline phase and each other to assess for variations. Data was analyzed via excel, MATLAB and a custom-built learning management and assessment platform (PREPARE), which enabled time synchronized collection of physiological data with data from manikins [14]. Data cleaning and standardization was performed by a combination of range-based and statistical methods. Outliers, possibly emerging from erratic equipment behavior, anomalies, or unique physiological reactions of participants, were identified and discarded to maintain data integrity. Phase 3 did not last for the intended three minutes. To evenly compare the phases the first two minutes of each phase was used for data analysis. Physiological measures were standardized across subjects via an algorithm that derived percentage deviations of measures from baseline [14]. This approach ensured that subsequent analyses were anchored to each participant's resting physiological state. By grounding the analyses in each participant's physiological norm, genuine physiological shifts induced by the various stressors can be identified, effectively sidelining inherent inter-participant variance.

## Results

On average, subjects provided a shorter duration of ventilations than recommended by the AHA. The average number of ventilations in all phases exceeded the goal of 10 per minute, with the highest average observed during the stress phase (16 ventilations per minute). Interestingly, Subject 4 provided >20 ventilations per minute during the baseline and stress phase. The PIP delivered by all subjects was within the parameters during the baseline phase. During the stress and distraction phase, the PIP delivery increased to 24.4 cmH^2^O/mL and 22.3 cmH_2_O/mL respectively. Of note, Subjects 1 and 2 provided increasing PIP as they progressed through the phase of the scenario, while Subject 3 consistently provided a PIP within the parameters (Table 1). 

**Table 1:** BVM performance Phase 1 baseline portion of simulation. Phase 2 stress portion of simulation. Phase 3 distraction portion of simulation. BVM ventilations expectations one ventilation delivered over one second every six seconds. BVM: Bag-valve-mask; PIP: Peak inspiratory pressure

Average duration of ventilations per phase (seconds)	Phase 1	Phase 2	Phase 3
Subject 1	0.58	0.49	0.58
Subject 2	0.61	0.85	0.85
Subject 3	0.57	0.64	0.59
Subject 4	0.64	0.73	0.51
Averages	0.6	0.68	0.63
Average number of ventilations provided per phase (per minute)			
Subject 1	9.5	14.5	11
Subject 2	9	12.5	11.5
Subject 3	11	15.5	11
Subject 4	20.5	21.5	14
Averages	12.5	16	11.9
Average PIP delivered (cmH_2_O/mL) per phase			
Subject 1	16.5	28.1	34.1
Subject 2	16.1	26.7	30.6
Subject 3	11.1	14.8	11.8
Subject 4	18.3	28.1	12.6
Averages	15.5	24.4	22.3

**Figure 2:**
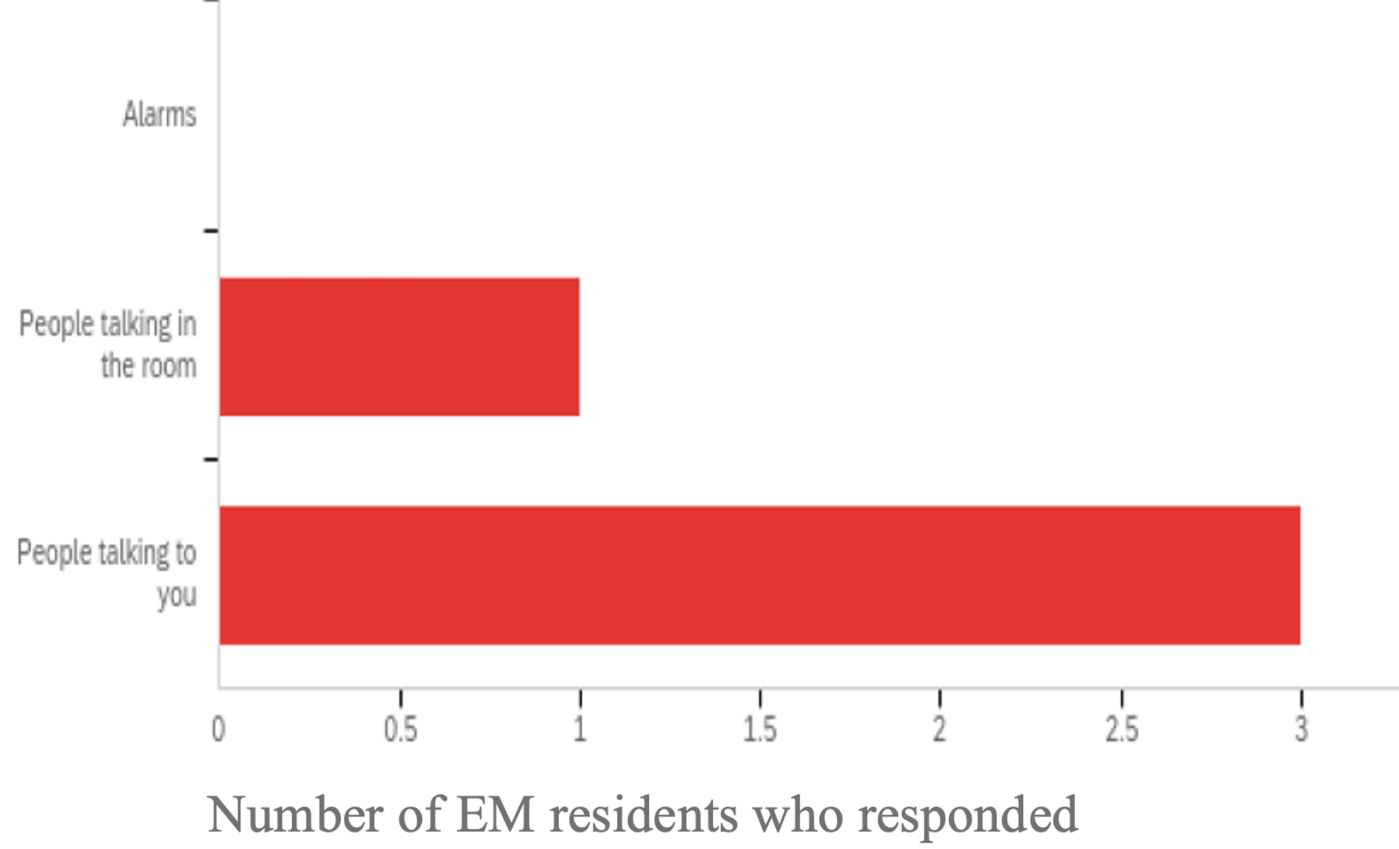
Post simulation survey results on self-identified impact on performance EM: Emergency medicine

The physiologic data showed all subjects' experiences increased percent deviations from their baseline during both the stress and distraction phase (Table 2). Three of the four subjects demonstrated a slightly greater percent deviation in HR during the distraction phase compared to the stress phase. While three of the subjects experienced greater then 20% deviation during both phases, Subject 2 showed less than a 20% deviation during both stages. The EDA data showed that three of the four subjects had a large percent deviation during the distraction phase compared to the stress phase. Interestingly, Subject 3 experienced 128.3% and 190.3% deviation from baseline during the stress and distraction phase respectively. Subject 2 also had a 113.6% deviation during the distraction phase. The post simulation survey results show all four subjects identified the distraction phase as the most impactful on their performance (Figure 2).

**Table 2:** Comparative physiological responses: HR and EDA HR: Heart rate; EDA: Electrodermal activity

Subject ID	Phase	HR percent deviation from baseline	EDA percent deviation from baseline
Subject 1	Stress	27.6	25
	Distraction	29.8	63.9
Subject 2	Stress	16.9	64.3
	Distraction	18.4	113.6
Subject 3	Stress	38.5	128.3
	Distraction	34.9	190.3
Subject 4	Stress	22.6	62.6
	Distraction	23.1	57.6

## Discussion

The results obtained from each phase of our study unveiled intriguing trends that both substantiated our initial hypothesis and aligned with prior literature. During the stress phase, we observed the highest average number of ventilations delivered and PIP provided. These results support the first part of our hypothesis that stress would cause an increase in the number of ventilations. Interestingly, the physiologic data showed on average subjects had a greater percent deviation from base line of both HR and EDA during the distraction phase compared to the stress phase. While the distraction phase showed greater change, the results still indicate that our subjects were under physiologic stress which may provide a reason for the increased ventilatory rate and PIP delivered. These results also support prior literature studying BVM performance demonstrated that HCP frequently hyperventilate and provide too much tidal volume [17-20]. 

The distraction phase showed a global reduction in the number of ventilations delivered compared to the stress phase, but conflicting results for the PIP delivered. Two subjects provided higher PIP during the distraction phase compared to the stress phase and two had decreased PIP provided. Distractions could have caused reduced attention and as such the reduced ventilation rate observed. This result supports the second part of our hypothesis that distraction would lead to less ventilations delivered. The EDA and HR data reveled subjects on average were experiencing greater stress during the distraction phase. Two subjects even had >100% deviation of EDA from base line during the distraction phase. All subjects indicated that the distraction phase was the most impactful on them and that is supported by their physiologic data. However, their performance results indicated that stress had the greatest impact on performance. This result opens the door to using feedback deceives to further studying perceived versus actual performance decrements.

The observations made from this study support the abundance of the current literature available in this arena. BVM performance studies demonstrate that HCPs frequently hyperventilate and provide too much tidal volume [17-20]. We had similar results, but also were able to appreciate that different stimuli induced different performance responses. This finding coincides nicely with the research of others looking at various stressors and their impact on task engagement and performance [24-26]. We also had several individual responses that showed subjects having performance decrements at baseline or were more susceptible to certain stimuli. These results lend themselves to the idea of individual performance profiles to train their weaknesses, an idea already being trialed by the military [23].

Limitations 

This study encountered several limitations with the primary concern being the small sample size, which was exacerbated by having to exclude two subjects due to technical and recording issues. This small sample size limited the ability to perform in-depth statistical analyses. As a result, we sought to identify trends that could spark future research in this area. The nature of testing stress and distraction sequentially could have confounded the results because subjects were not at baseline at the start of the distraction phase. What was observed has the potential to be a stacking effect and further studies would benefit from testing these individually. Like all simulations, our scenario does not replicate real-life situations, which may lead to results that do not reflect reality. The implementation of wearable physiologic monitors during clinical activities could provide more comprehensive and accurate data regarding stressor and distraction. 

## Conclusions

This study demonstrated that stress and distraction lead to a change in subjects physiologic state which provides an answer for their BVM ventilation performance decrements. HR variability (HRV), EDA and high-fidelity manikins are valuable feedback devices that if able should be implemented in all simulation training to assessing subjects stress state and technical performance. A future study with a larger sample size could better reveal performance and physiologic trends amongst subjects and the impact of different stimuli. This information could identify when a provider may be at-risk for performance decrement and guide simulation training to allow for peak performance during patient care.

## References

[1] Wrenn K, Lorenzen B, Jones I, Zhou C, Aronsky D (2010). Factors affecting stress in emergency medicine residents while working in the ED. Am J Emerg Med.

[2] Jämsä JO, Uutela KH, Tapper AM, Lehtonen L (2021). Clinical alarms and alarm fatigue in a university hospital emergency department-a retrospective data analysis. Acta Anaesthesiol Scand.

[3] LeBlanc VR (2009). The effects of acute stress on performance: implications for health professions education. Acad Med.

[4] Babapour AR, Gahassab-Mozaffari N, Fathnezhad-Kazemi A (2022). Nurses' job stress and its impact on quality of life and caring behaviors: a cross-sectional study. BMC Nurs.

[5] Suryavanshi N, Kadam A, Dhumal G (2020). Mental health and quality of life among healthcare professionals during the COVID-19 pandemic in India. Brain Behav.

[6] Ratwani RM, Fong A, Puthumana JS, Hettinger AZ (2017). Emergency physician use of cognitive strategies to manage interruptions. Ann Emerg Med.

[7] Westbrook JI, Raban MZ, Walter SR, Douglas H (2018). Task errors by emergency physicians are associated with interruptions, multitasking, fatigue and working memory capacity: a prospective, direct observation study. BMJ Qual Saf.

[8] Cook DA, Hatala R, Brydges R (2011). Technology-enhanced simulation for health professions education: a systematic review and meta-analysis. JAMA.

[9] Ilgen JS, Sherbino J, Cook DA (2013). Technology-enhanced simulation in emergency medicine: a systematic review and meta-analysis. Acad Emerg Med.

[10] Salik I, Paige JT (2023). Debriefing the interprofessional team in medical simulation. https://www.ncbi.nlm.nih.gov/books/NBK554526/.

[11] Coleman JJ, Robinson CK, von Hippel W (2023). What happens on call doesn't stay on call. The effects of in-house call on acute care surgeons' sleep and burnout: results of the Surgeon Performance (SuPer) trial. Ann Surg.

[12] Abahuje E, Reddy S, Rosu C (2023). Relationship between residents' physiological stress and faculty leadership skills in a department of surgery. J Surg Educ.

[13] Janicki AJ, Frisch SO, Patterson PD, Brown A, Frisch A (2020). Emergency medicine residents experience acute stress while working in the emergency department. West J Emerg Med.

[14] Pappada S, Owais MH, Aouthmany S (2022). Personalizing simulation-based medical education: the case for novel learning management systems. Int J Healthcare Simul.

[15] Clarke S, Horeczko T, Cotton D, Bair A (2014). Heart rate, anxiety and performance of residents during a simulated critical clinical encounter: a pilot study. BMC Med Educ.

[16] Panchal AR, Bartos JA, Cabañas JG (2020). Part 3: adult basic and advanced life support: 2020 American Heart Association guidelines for cardiopulmonary resuscitation and emergency cardiovascular care. Circulation.

[17] Merrell JG, Scott AC, Stambro R, Boukai A, Cooper DD (2023). Improved simulated ventilation with a novel tidal volume and peak inspiratory pressure controlling bag valve mask: a pilot study. Resusc Plus.

[18] Dafilou B, Schwester D, Ruhl N, Marques-Baptista A (2020). It's in the bag: tidal volumes in adult and pediatric bag valve masks. West J Emerg Med.

[19] Sall FS, De Luca A, Pazart L, Pugin A, Capellier G, Khoury A (2018). To intubate or not: ventilation is the question. A manikin-based observational study. BMJ Open Respir Res.

[20] O'Neill JF, Deakin CD (2007). Do we hyperventilate cardiac arrest patients?. Resuscitation.

[21] Silva PL, Rocco PR (2018). The basics of respiratory mechanics: ventilator-derived parameters. Ann Transl Med.

[22] Mumma JM, Durso FT, Dyes M, Dela Cruz R, Fox VP, Hoey M (2017). Bag valve mask ventilation as a perceptual-cognitive skill. Hum Factors.

[23] Martin K, Périard J, Rattray B, Pyne DB (2019). Physiological factors which influence cognitive performance in military personnel. Hum Factors.

[24] Matthews G (2016). Multidimensional profiling of task stress states for human factors: a brief review. Hum Factors.

[25] Matthews G, Campbell SE, Falconer S (2002). Fundamental dimensions of subjective state in performance settings: task engagement, distress, and worry. Emotion.

[26] Joseph M, Ray JM, Chang J (2022). All clinical stressors are not created equal: differential task stress in a simulated clinical environment. AEM Educ Train.

